# Characteristics and influencing factors of amyloid fibers in *S. mutans* biofilm

**DOI:** 10.1186/s13568-019-0753-1

**Published:** 2019-02-28

**Authors:** Dongru Chen, Yina Cao, Lixia Yu, Ye Tao, Yan Zhou, Qinghui Zhi, Huancai Lin

**Affiliations:** Department of Preventive Dentistry, Guanghua School of Stomatology, Guangdong Provincial Key Laboratory of Stomatology, Sun Yat-sen University, Guangzhou, Guangdong China

**Keywords:** Amyloid fibers, *S. mutans*, biofilm, eDNA

## Abstract

There are signs that amyloid fibers exist in *Streptococcus mutans* biofilm recently. However, the characteristics of amyloid fibers and fibrillation influencing factors are unknown. In this study, we firstly used transmission electron microscopy (TEM) and atomic force microscopy (AFM) to observe the morphology of amyloid fibers in *S. mutans*. Then the extracted amyloid fibers from biofilm were studied for their characteristics. Further, the influencing factors, PH, temperature and eDNA, were investigated. Results showed there were mainly two morphologies of amyloid fibers in *S. mutans*, different in width. Amyloid fibers inhibitor-EGCG obviously destroyed biofilm at different stages, which is dose-dependent. The amount of amyloid fibers positively correlated with biofilm biomass in clinical isolates. Acidic pH and high temperature obviously accelerated amyloid fibrillation. During amyloid fibrillation, amyloid growth morphologies were observed by TEM and results showed two growth morphologies. Amyloid fibers formed complex with eDNA, which we call (a)eDNA. The molecular weight of (a)eDNA was similar to genomic DNA, greatly larger than that of eDNA in matrix. Combined use of DNase I and EGCG was more efficiently in inhibiting amyloid fibers and biofilm biomass. In conclusion, amyloid fibers are the crucial structures for *S. mutans* biofilm formation, showing two types of morphology. Acidic pH and temperature can obviously accelerate amyloid fibrillation. Amyloid fibers form complex with (a)eDNA and combined use of DNase and amyloid fiber inhibitor is more efficiently in inhibiting *S. mutans* biofilm formation.

**Electronic supplementary material:**

The online version of this article (10.1186/s13568-019-0753-1) contains supplementary material, which is available to authorized users.

## Introduction

It is revealed that among all microbial and chronic infections, 65% and 80% are associated with biofilm formation, respectively (Jamal et al. 2018). Recently, amyloid fibers are found in the surface of bacteria and play important role in biofilm formation. Unlike misfolded toxic aggregates in human tissues, amyloid fibers formed on the surface of bacteria can provide biological functions, thus be called functional amyloid fibers (Erskine et al. 2018). In bacteria, amyloid fibers often mediate cell–cell and cell–surface interactions. Besides, amyloid fibers can act as protection barrier or interfere with the function of specific proteins, and amyloid fibers may thus represent novel targets for antibacterial drugs (Blanco et al. 2012).

The list of bacteria with well-characterized, amyloid-forming proteins is growing (Erskine et al. 2018). The foremost known biofilm-associated functional amyloid fibers are the Curli fibers from Gram-negative *Escherichia coli* (*E. coli*) (Chapman et al. 2002; Hammar et al. 1995). *E. coli* has a dedicated secretion system, chaperone proteins and inhibitors. More bacteria with dedicated secretion systems forming amyloid fibers have been described, e.g. various species of *Pseudomonas* (Dueholm et al. 2013). Unlike *E. coli* with dedicated secretion systems, cell surface proteins on some bacteria have been discovered to form amyloid fibers. Cell surface proteins are reported to build amyloid structures only under some specific environmental conditions, such as low pH, high temperature, metal ion (Taglialegna et al. 2016a). Beside environmental factors, studies indicate that biofilm matrix components, extracellular DNA (eDNA), may accelerate amyloid fibrillation and form complex with amyloid fibers (Gallo et al. 2015; Schwartz et al. 2016). But the information about eDNA and amyloid fibers complex is limited and more studies should be investigated.

biofilm formation by *Streptococcus mutans* (*S. mutans*) is considered as the initial and crucial virulence factor causing dental caries (Khan et al. 2011). Recently, there are signs that *S. mutans* have amyloid fibers and using epigallocatechin gallate (EGCG), an inhibitor of amyloid fibrillation, can decrease ThT fluorescence intensity of *S. mutans* biofilm (Oli et al. 2012). P1, WapA, and SMU_63c have been reported to form amyloid fibers, among which P1 and WapA are the surface proteins associated with biofilm formation, while SMU_63c is an uncharacterized secreted protein (Besingi et al. 2017; Tang et al. 2016). Truncated proteins C123 (aa 1000-1486) of P1 and AgA (aa 30-323) of WapA are identified to auto-aggregated into amyloid fibers under neutral pH by stirring in vitro (Besingi et al. 2017). However, amyloid fibers’ characteristics and fibrillation influencing factors in *S. mutans* has not ever been investigated. The natural habitat of *S. mutans* is human oral cavity, and oral cavity is a dynamic environment that undergoes large and rapid fluctuations in pH, nutrient availability, oxygen tension and temperature (Lemos et al. 2005). These environmental factors might influence amyloid fibrillation and *S. mutans* biofilm formation. In our study, we aim to investigate the important role of amyloid fibers at different stages during *S. mutans* biofilm formation, and to verify whether amyloid fibers are the universal structure in clinical isolates in biofilm formation and whether amyloid fibers appear in planktonic state. Moreover, we would extract the amyloid fibers from *S. mutans* biofilm and obtain aggregated amyloid fibers through purified C123 to study their characteristics and influencing factors. Our findings would provide theoretical basis for inhibiting *S. mutans* biofilm formation by influencing amyloid fibrillation.

## Materials and methods

### biofilm formation, crystal violet and ThT assay

Planktonic *S. mutans* UA159 (ATCC^®^ 700610™) was cultured in brain heart infusion broth media (BHI). The 1:100 diluted plateau stage planktonic *S. mutans* was cultured in BHI with 1% sucrose (BHIs) at 37 °C for biofilm formation. At each time point, plates were taken out for crystal violet and ThT assay. For EGCG or DNase I treated biofilm formation, BHIs were added by EGCG at a final concentration of 50 μM, 100 μM or 200 μM, or added by DNase I at a final concentration of 2 U/μl. biofilm was washed by PBS for two times, fixed by formaldehyde for 15 min, air dried for 15 min, stained by 0.1% crystal violet for 15 min, washed by ddH_2_O until no excess dye, and then the 96-well plate was measured by spectrophotometer for OD_600_. For ThT assay, *S. mutans* was cultured in opaque 96-well plates with flat clear bottom. After washing, biofilm was stained by ThT for 30 min, at a final concentration of 20 μM. The fluorescence intensity of ThT was measured at 25 °C by multifunctional spectrophotometer (SpectraMax M5) with excitation at 430 nm, emission at 490 nm and a cut-off at 475 nm.

### Confocal laser scanning microscopy (CLSM)

biofilm was stained by 1 μg/ml SYTO9 for live bacteria and 1 μg/ml propidium iodide for dead bacteria. Besides, biofilm were stained by 1 μg/ml SYTO9 for live bacteria, 10 μg/ml CR, and 1 μg/ml TOTO-1 to observe the position relationship among *S. mutans*, amyloid and eDNA (Gallo et al. 2015). After staining, CLSM images were taken.

### Transmission electron microscopy (TEM)

Formvar/carbon-coated nickel grids were deposited by a drop of fixed *S. mutans*, scraping from biofilm, for 2 min, and negative stained for 2 min by using 3% phosphotungstic acid, and then washed by ddH_2_O for a few seconds. Observations were made with a Hitachi TEM system.

### Atomic force microscopy (AFM)

Fixed *S. mutans* was deposited on the surface of freshly cleaved mica and left to dry in air at room temperature. Images were obtained using a Nanoscope IIIa Multimode control system (Dimension Fastscan, Bruker) operating in tapping mode.

### Isolation of amyloid fibers

The modified method of amyloid fibers isolation were based on two methods described by Schwartz et al. (2012) and Romero et al. (2010). biofilms were grown in eight 10 cm^2^ dishes for 24 h. After washing by PBS for two times, biofilms were scraped and diluted in 1 ml/dish saline extraction buffer (5 mM potassium phosphate, 2 mM MgCl2, 100 mM morpholi nepropane sulphonic acid (Mops) and 1 M NaCl, PH = 7) supplemented with a protease inhibitor mixture. The biofilm suspensions were homogenized using a tissue homogenizer (TissueMiser, Fisher) to shear fibers free from the cell walls. Supernatants were clarified by repeated centrifugation (seven times) at 7000 rpm for 2 min to remove bacteria. At last, supernatants without cells were centrifugation at 16,000*g* for 20 min to precipitate amyloid fibers, and the precipitate was redissolved in distilled deionized water. Presence of fibers was confirmed via TEM imaging. Amyloid fibers solution was treated by DNase I, RNase and protease K. After treating, amyloid fibers were confirmed by TEM.

### Protein expression and purification

The truncated protein C123 of P1 (amino acids 1000-1486) was PCR amplified from *S. mutans* UA159 genomic DNA. Fragments were cloned in pET-30a(+) vector. Overnight cultures of Escherichia coli BL21(DE3) containing plasmids were diluted 1:100 and grown to an OD_600_ nm of 0.6. Isopropyl B-d-thiogalactopyranoside (IPTG) was added with the final concentration of 0.5 mM for induction at 15 °C for 16 h and at 37 °C for 4 h, respectively. Pellets were resuspended with lysis buffer followed by sonication and centrifugation. Protein was obtained by two-step purification using Ni column and Superdex 200 Column and sterilized by 0.22 μm filter. The concentration was determined by Bradford protein assay with BSA as a standard. The protein purity and molecular weight were determined by standard SDS-PAGE along with western blot confirmation.

### Aggregation of purified truncated proteins

Purified truncated protein of C123 was diluted in ddH_2_O with PH = 7 or PH = 3 at 37 °C or 60 °C. 100 μl aggregated protein solution mixed with 200 uM ThT were monitored for amyloid fiber growth kinetics. The final protein concentration was 0.1 mg/ml.

### Extraction of (a)eDNA from amyloid fibers

Amyloid solution was treated by equal volume of absolute ethyl alcohol and 1/10 volume of the DNA extraction buffer (3 M sodium acetate containing 1 mM EDTA, PH = 5.2) to extract (a)eDNA.

### Extraction of genomic DNA and eDNA from biofilm

*Streptococcus mutans* genomic DNA was extracted by using Bacteria DNA Kit (OMEGA, D3350-01, USA). The extraction of eDNA was referred to Liao et al. (2014).

## Results

### Morphology of amyloid fibers in *S. mutans*

**Fig. 1:**
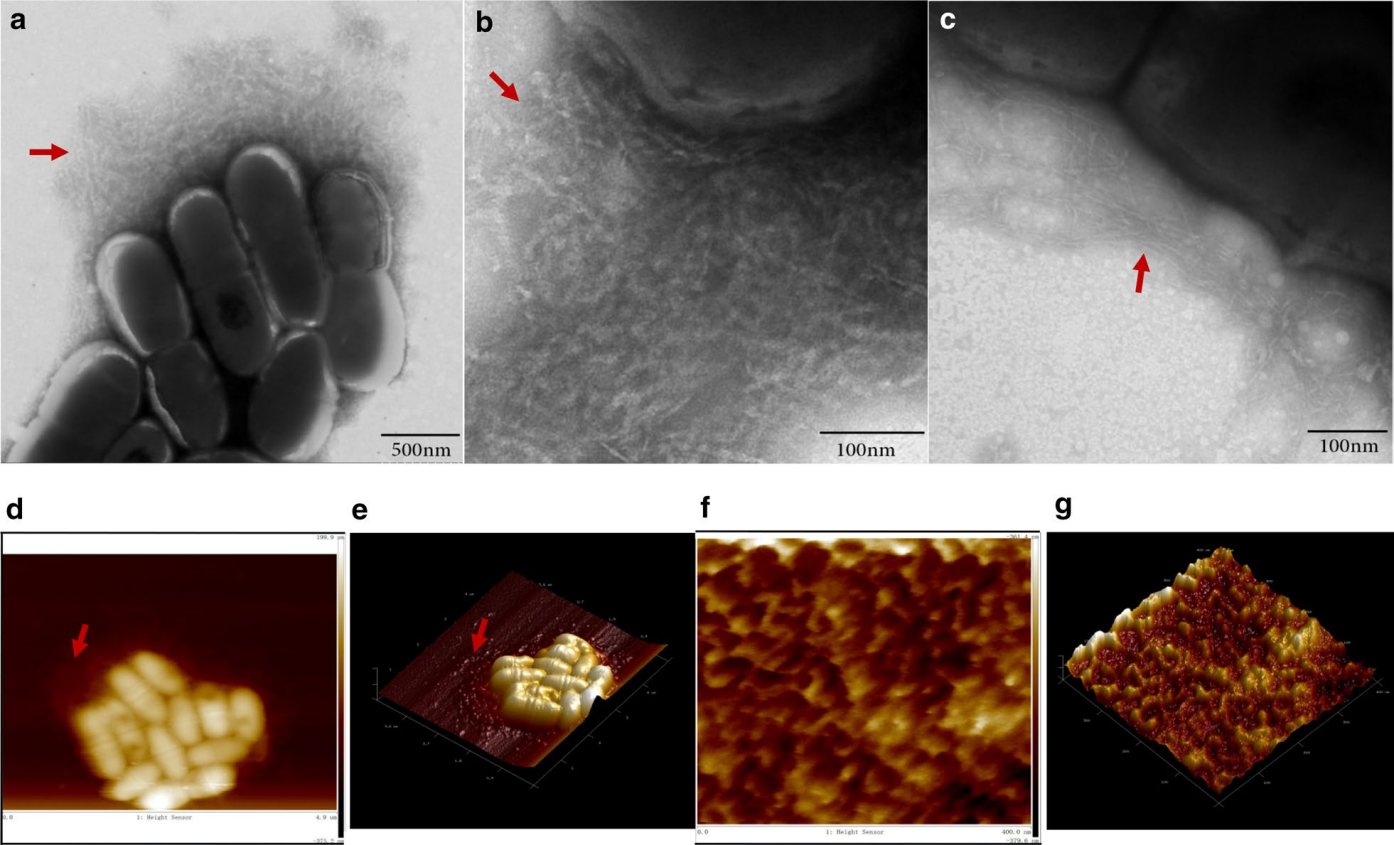
Images of amyloid fibers in *S. mutans* taken by TEM and AFM. **a** TEM image showed amyloid fibers on *S. mutans* in biofilm. **b** Image showed the amplified thick amyloid fibers. **c** Image showed the amplified thin amyloid fibers. **d** Height sensor AFM image showed amyloid fibers in *S. mutans* in biofilm. **e** Three-D reconstruction AFM image showed the amyloid fibers emanating from amyloid fibers. **f** Amplified height sensor image showed the crossed amyloid fibers in *S. mutans*. **g** Three-D reconstruction of the amplified image showed crossed amyloid fibers in *S. mutans.* Red arrows: Amyloid fibers on *S. mutans*

When observed by TEM, amyloid fibers emanating from the *S. mutans* could be observed, which showed various length, from 50 nm to several microns (Fig. 1a). Generally, amyloid fibers on *S. mutans* had two morphologies, different in width. The thick one was fuzzy and about 10–16 nm, (Fig. 1b), while the thin one was clear and about 4-6 nm (Fig. 1c). Height image (Fig. 1d) and 3D reconstruction image (Fig. 1e) of amyloid fibers in *S. mutans* taken by AFM were supplied. The amplified images of amyloid fibers (Fig. 1f, g) in *S. mutans* suggested that amyloid fibers intertwined with each other and formed like net in vitro.

### Amyloid fibers promoting *S. mutans* biofilm formation

**Fig. 2:**
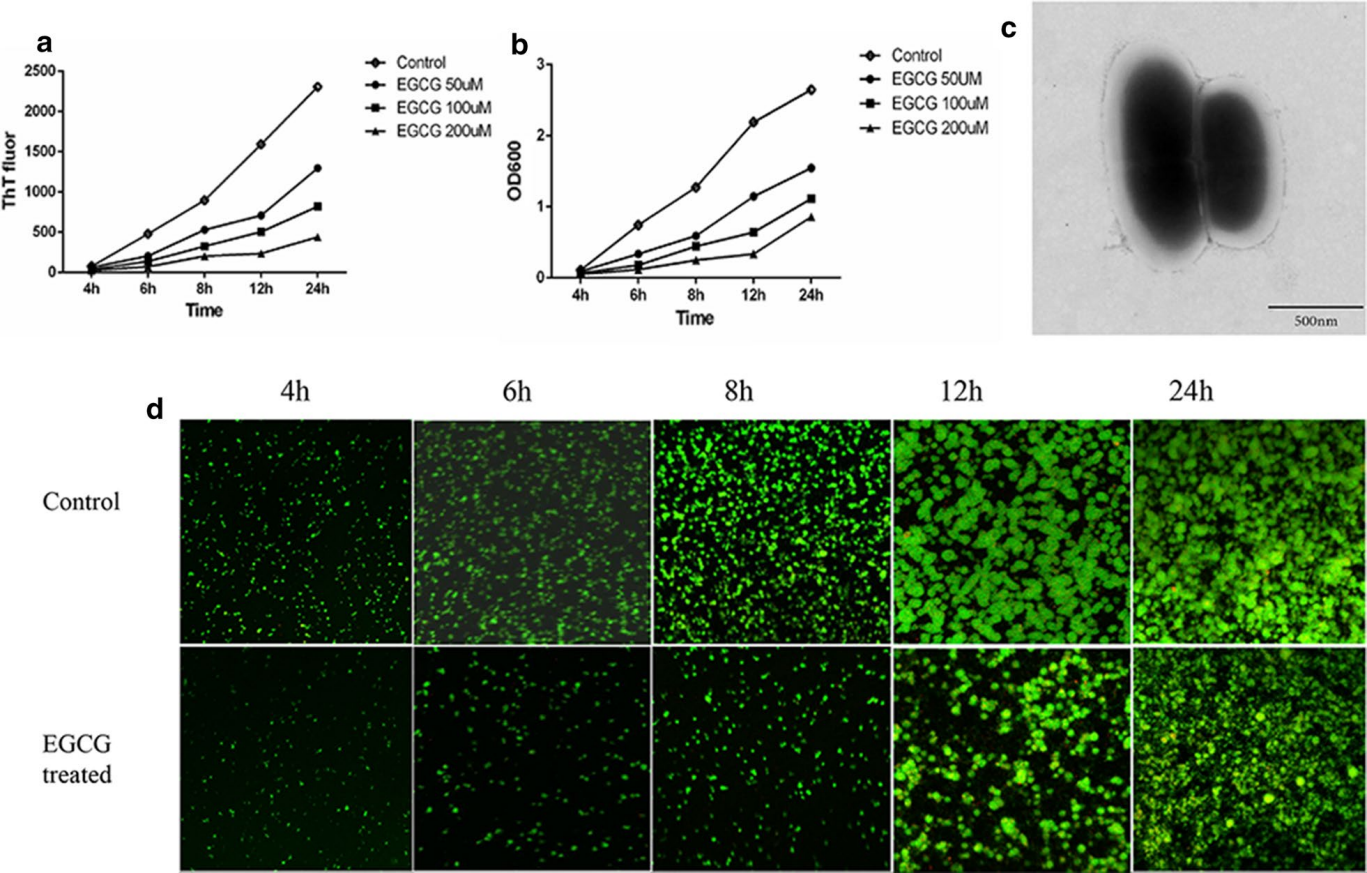
Results of EGCG treated. **a** The ThT fluorescence intensity of amyloid fibers at different time during biofilm formation, with EGCG treated or not (n = 3). EGCG can significantly reduce the amount of amyloid fibers at different times during *S. mutans* biofilm formation. **b** The biofilm biomass at different time during biofilm formation, with EGCG treated or not (n = 3). EGCG can significantly reduce the amount of amyloid fibers with dose-dependent. **c** TEM image of the washed away *S. mutans* in biofilm with EGCG treated. **d** Live/dead staining images of the biofilm at different time, with EGCG treated or not

During biofilm formation, the ThT fluorescence intensity shared similar pattern with that of biofilm biomass. EGCG at 50 μM, 100 μM and 200 μM obviously decreased the amount of amyloid fibers and biofilm biomass at all biofilm growth stages, with a dose-dependent decrease (Fig. 2a, b). We found biofilm was fragile and easy to be washed away when treated by EGCG. We collected the washed away bacteria and amyloid fibers could rarely be observed under TEM (Fig. 2c), which indicated that amyloid fibers were the crucial structure for biofilm formation and integrity. Besides, the live/dead bacteria staining also indicated that biofilm was obviously decreased when treated by EGCG, in contrast to the untreated group (Fig. 2d).

### Amyloid fibers were the universal structure in clinical isolates

**Fig. 3:**
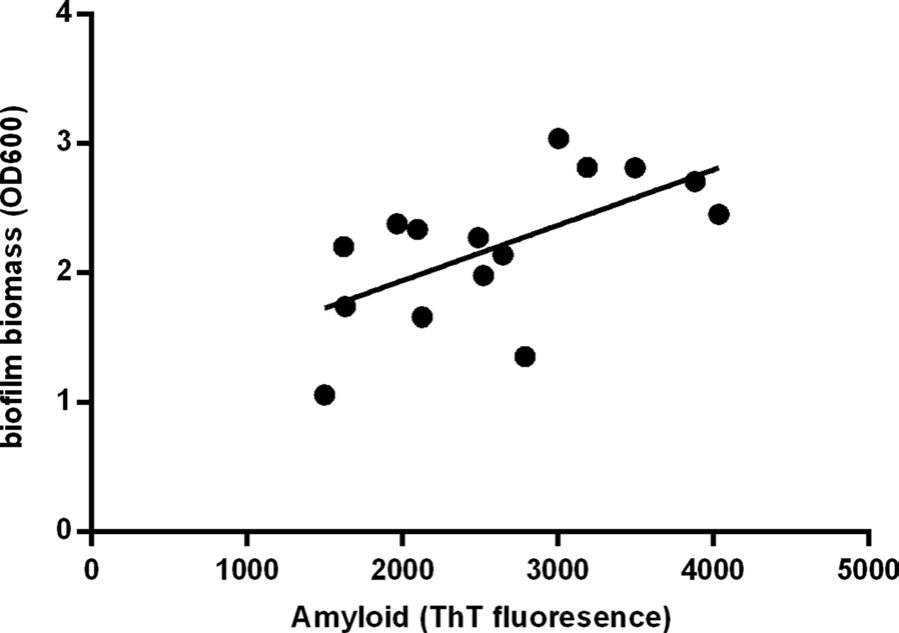
Correlation of ThT fluorescence intensity of amyloid fibers and biofilm biomass (OD_600_). Results showed significant correlation (R square = 0.3774, *p *= 0.0148)

To observe whether amyloid fibers universally exist in the clinical isolates of *S. mutans* and correlated with their biofilm formation, we randomly selected 15 clinical isolates of *S. mutans* from our previously separated clinical isolates (Zhou et al. 2018). We cultured these clinical isolates for 24 h for biofilm formation, and found ThT fluorescence intensity had a significant linear correlation with biofilm biomass (*p *< 0.05; Fig. 3), which demonstrated that amyloid fibers were also correlated with biofilm formation in clinical *S. mutans* isolates, and amyloid fibers were the universal structure for biofilm formation.

### Few amount of amyloid fibers existed in planktonic *S. mutans*

Next, to learn whether amyloid fibers only appear in biofilm, we explored whether planktonic *S. mutans* had amyloid fibers. When planktonic *S. mutans* grew to the saturation period, cells were centrifuged and observed under TEM, we also found few cells had amyloid fibers, but they showed very small ratio compared with that in biofilm (Additional file 1: Figure S1A). However, planktonic *S. mutans* had another connection structure between cells, with large amount, also emanating from the cell surface, but not like amyloid fibers when observed by TEM (Additional file 1: Figure S1B).

### Isolation and characterization of amyloid fibers

In order to know more about the characteristics of amyloid fibers, we isolated amyloid fibers from cells. Successful isolation of amyloid fibers was verified by TEM (Additional file 1: Figure S2A). Amyloid fibers without treatment were run by SDS-page, and no bands could be seen (Additional file 1: Figure S2B), which was in consistent with the characteristics that amyloid fibers could dissolve in SDS. Moreover, we treated the extracted amyloid fibers with protease K, DNase I and RNase. After treated, amyloid fibers could still be detected by TEM (Additional file 1: Figure S2C).

### Characteristics of amyloid fibers aggregated by purified proteins and fibrillation influencing factors

**Fig. 4:**
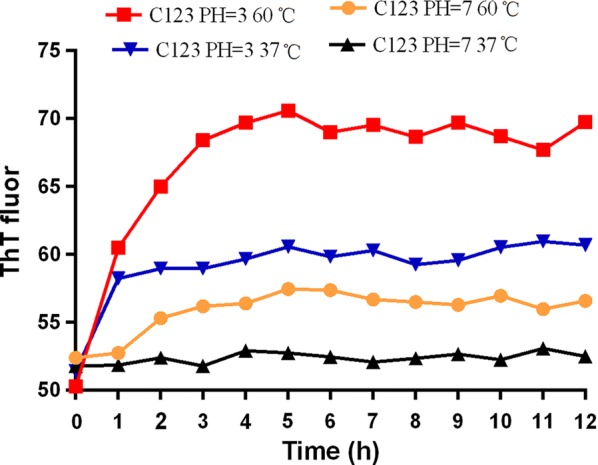
The kinetics of the purified C123 aggregation, with different PH and temperature. Results showed that acidic PH and high temperature could accelerate C123 aggregation

To learn the factors influencing amyloid fibrillation, the already known amyloid forming proteins, truncated protein C123 was purified for aggregation. C123 had a long lag time when observed at 37 °C, PH = 7 (Fig. 4), and until 48 h a small amount of amyloid fibers be observed by TEM. However, amyloid fibers could already be detected after culture at 37 °C for 4 h in biofilm formation, which meant that amyloid fibrillation was much rapid in vivo. ThT fluorescence results showed that low PH (PH = 3) obviously accelerated the process of amyloid fibrillation, and high temperature (60 °C) could also increase the process (Fig. 4). Acidic PH and high temperature both reduced the lag times, and the matured amyloid fibers could be observed at 4 h or 5 h after culture.

**Fig. 5:**
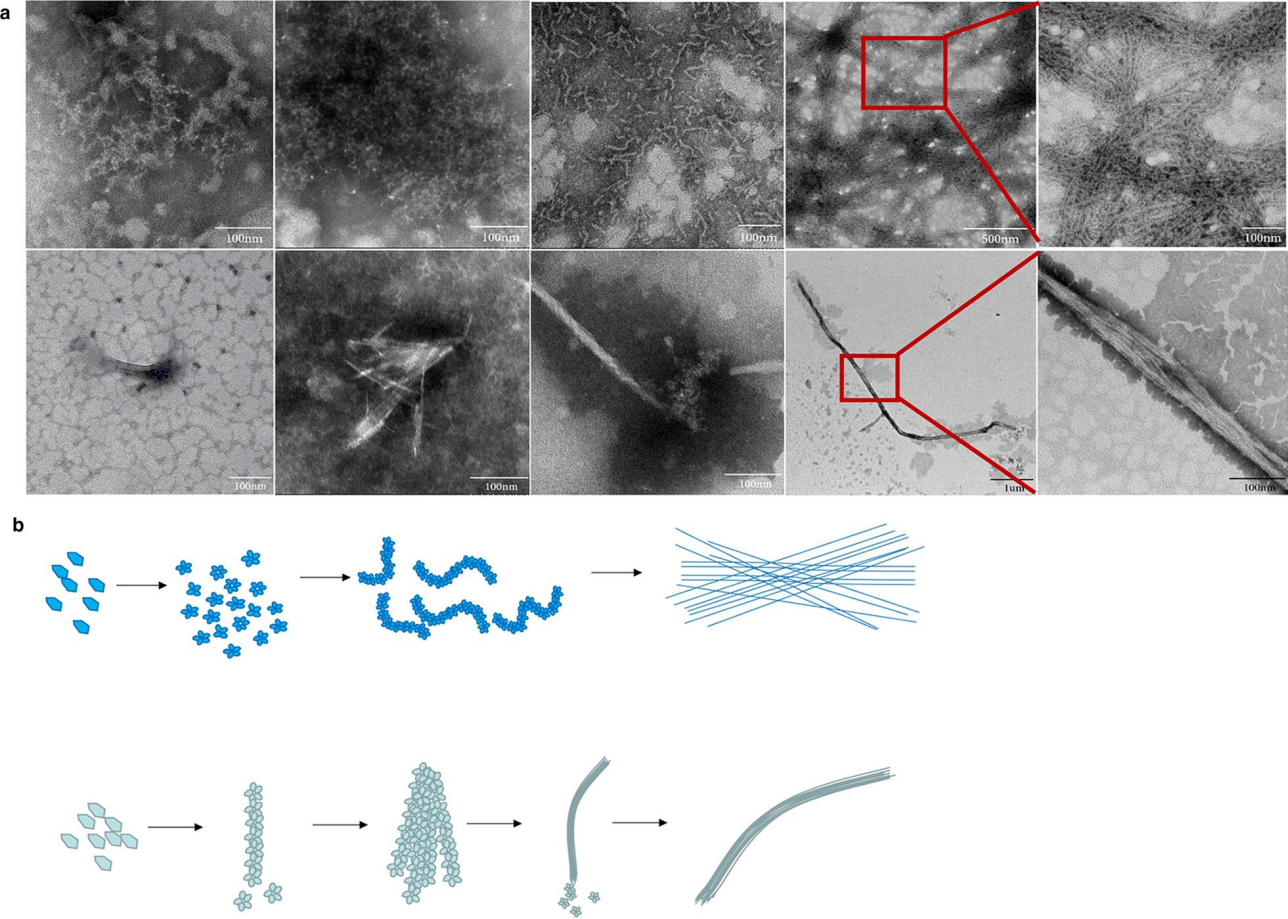
The two types of the purified C123 aggregation process at different times. **a** TEM images showing the two aggregation types. Red arrows: The intermediate products of C123 aggregation. **b** The mode patterns of the two aggregation types

At the same time, the aggregated amyloid fibers at different phases were observed by TEM, and two aggregated types could be found (Fig. 5a). Type I was the dominating one, firstly few disordered granular structures could be seen, and these structures multiplied gradually. Then large amount of twisted short rod-like structures, about 4–6 nm in width and 20–100 nm in length, were observed. Finally, intertwined rigid amyloid fibers were seen. Type II could also be seen, but with relatively smaller amount. Firstly, small granular particles appeared and then formed into relatively straight short rods, after that several rods would unite together. Gradually, amyloid fibers grew longer by recruiting more particles to one tail of the fiber. In the end, they compacted into thick matured amyloid fibers with various lengths. The aggregated amyloid fibers formed by purified C123 truncated protein were different from amyloid fibers on *S. mutans* in morphology, and the aggregated fibers were rigid while the amyloid fibers on *S. mutans* were “soft”. The above two mode aggregated patterns were provided for better interpretation (Fig. 5b).

### Amyloid fibers forming complex with eDNA

**Fig. 6:**
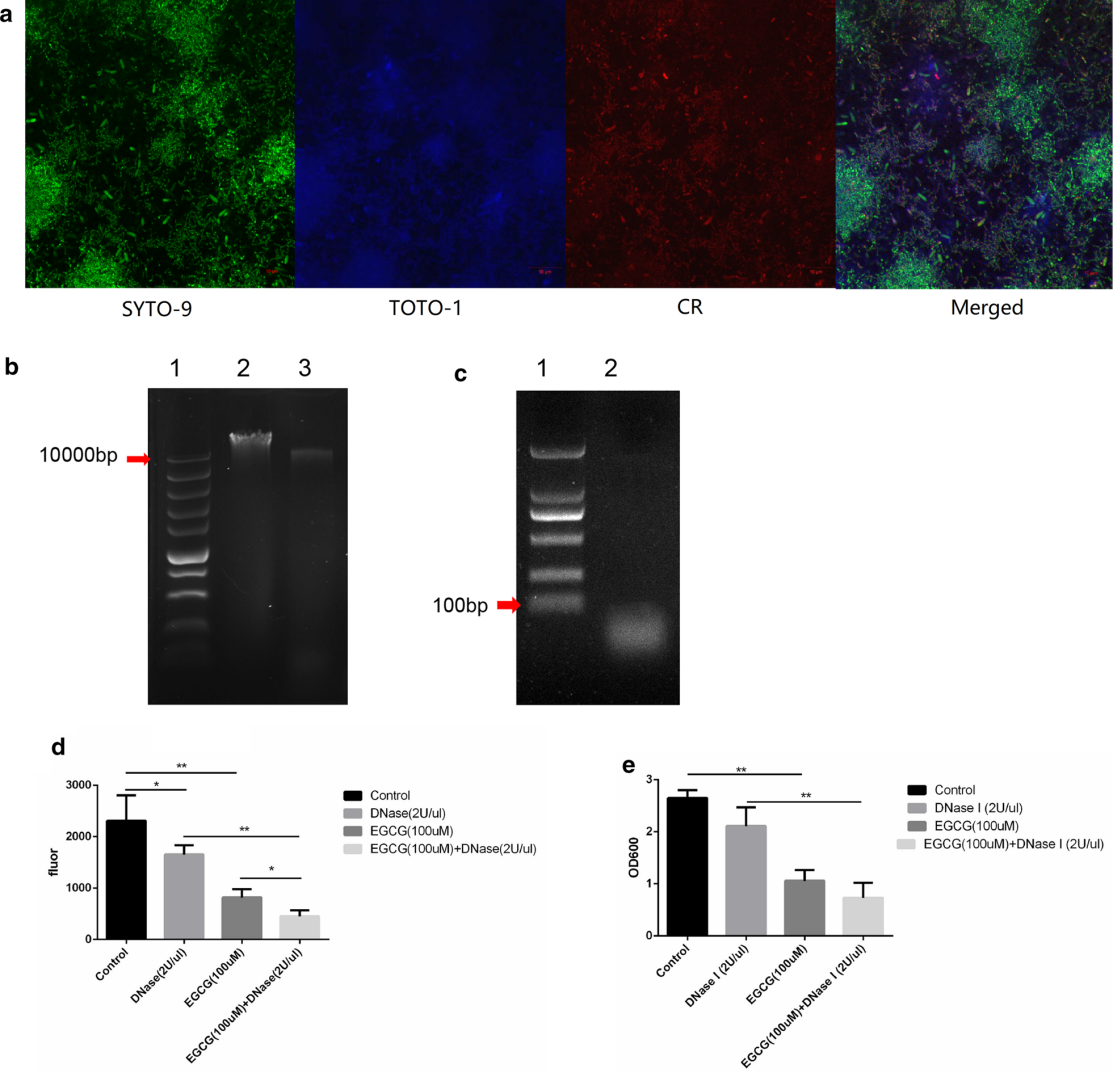
Relationship of amyloid fibers and eDNA. **a** LSCM images showing the position relationships among live bacteria (SYTO-9, green), amyloid fibers (CR, red) and eDNA (ToTo-1, blue). **b** AGE analysis of the *S. mutans* genomic DNA (lane 2) and (a)eDNA (lane 3). Lane 1 showed marker. **c** AGE analysis of the eDNA (lane 2). Lane 1 showed marker. **d** The ThT fluorescence intensity of amyloid fibers at 24 h, treated by DNase I or EGCG solely and together. **e** The biofilm biomass at 24 h, treated by DNase I or EGCG solely and together (n = 3, *p < 0.05, **p < 0.01)

Firstly, we investigated the position relationship among live bacteria, amyloid fibers and eDNA in the early biofilm through laser scanning confocal microscope (LSCM). Results showed that different amounts of amyloid fibers (red) were found around live bacteria, and a part of eDNA (blue) gathered around live bacteria (Fig. 6a), indicating that amyloid fibers and eDNA might colocalize in biofilm. But the staining might be false positive, thus more evidence was needed. To explore whether the extracted amyloid fibers had eDNA, we tried to extract eDNA from amyloid fibers, which we called (a)eDNA to distinguish from eDNA extracted directly from extracellular matrix. We successfully extracted (a)eDNA. Agarose electrophoresis (AGE) was used to assess the molecular weight differences, and results showed that the molecular weight (a)eDNA was smaller but approximate to genomic DNA (> 10,000 bp, Fig. 6b), much larger than that of eDNA (< 100 bp, Fig. 6c), which indicated that (a)eDNA be protected from degradation after forming complex with amyloid fibers.

After knowing that eDNA formed complex with amyloid fibers, we wondered whether DNase could reduce amyloid fibers and biofilm biomass in vivo. DNase and EGCG were added into BHIs solely or together when culturing biofilm. Results indicated that the amount of amyloid fibers was significantly reduced by adding DNase I alone (Fig. 6d), but no significant differences were found in biofilm biomass when adding DNase I alone (Fig. 6e). However, combined use of EGCG and DNase I was more efficient in inhibiting amyloid fibers and biofilm biomass (Fig. 6d, e).

## Discussion

In this study, we firstly show the clear morphology of amyloid fibers emanating from *S. mutans*, intertwined into net-like. The net formed by amyloid fibers around *S. mutans* might be helpful in gathering other matrix or cells. There are mainly two morphologies, different in width. The aggregated amyloid fibers by purified truncated protein C123 also follow two aggregation types, forming into rigid amyloid fibers. Various morphologies and properties of amyloid fibers produced by the same protein can also be found when exposed to differing environmental conditions during growth, including temperature, salt concentration and shear forces (Bekard and Dunstan 2009; Lee et al. 2007; Petkova et al. 2005; Yoshimura et al. 2012). Variations in fibril morphology with subtle changes in growth conditions can change the cytotoxicity of amyloid fiber (Petkova et al. 2005). However, the different roles of the two morphological different amyloid fibers in *S. mutans* are still unknown, which needs to be further explored.

Amyloid fibers are the crucial structures for both standard strain and clinical isolates in biofilm formation, and inhibiting amyloid fibers can destroy the stability of *S. mutans* biofilm during biofilm formation. Learning the characteristics and influencing factors of amyloid fibers in *S. mutans* biofilm would help us inhibit biofilm formation by targeting amyloid fibers. Amyloid fibrillation process often comprises three distinct phenomenological phases: a lag, growth and saturation phase (Blanco et al. 2012). The rate limiting step is the formation of the partially unfolded ensembles or nucleation in lag phase, and the therapeutic drugs to cure amyloid fibers associated diseases are to reduce lag time or inhibit nucleation. Many factors, including initial protein concentration, PH, temperature, ionic strength of the solution, seeding and the intensity of agitation, influence fibrillation process (Ow and Dunstan 2014). Acidic PH and high temperature are regarded as the good fibrillation condition (Sorci et al. 2009). Taglialegna et al. (2016b) found purified rBap_B protein forms aggregates when incubated at pH 4.5, while rBap_B aggregates disassemble when exchanging from PH 4.5 to PH 7.5. Many researches verify that purified proteins can aggregate into amyloid fibers at neutral PH but with a very long lag time, which is in accordance with our results (Gallo et al. 2015; Oli et al. 2012). Study also shows that the lag time becomes longer and the onset of fibril formation occurs later with cooling (25 °C) than without (65 °C). The reason of proteins at low temperature unable to form fibrils might be that the structural rearrangement step requires additional energy that is unavailable at 25 °C (Sorci et al. 2009). *S. mutans* is a kind of aciduric and thermotolerant stain, we assume that amyloid fibers produced by *S. mutans* under acidic and high-temperature conditions might be a protective mechanism, adapting themselves to harsh conditions.

Biofilm matrix, such as nucleic acid, proteins, carbohydrates and lipids, is also related with amyloid fibers, among which eDNA becomes the most popular studied one (Stewart and Radford 2017; Zhao et al. 2018). For eDNA derives from membrane vesicles at very early time during biofilm formation (Whitchurch et al. 2002). More importantly, eDNA also represent as nanofibers, ‘sweater-like’ mats or ‘yarn-like’ structures, which can act as scaffolds for proteins aggregation (Barnes et al. 2012; Liao et al. 2014). Schwartz et al. (2016) found conditions or mutants that did not generate eDNA resulted in lack of amyloids during biofilm growth despite the amyloidogeneic subunits being produced. Gallo et al. (2015) also found DNA accelerated the polymerization of *S. Typhimurium* amyloid curli in vitro, and amyloid curli and DNA colocalized within *S. Typhimurium* biofilm. It is suggested that DNA attracts the positively charged PSMs and raises the local peptide concentration, therefore resulting in polymerization (Payne and Boles 2016). In our study, we discovered various intensity of amyloid fibers stained by CR around live bacteria, and eDNA could also share similar position with amyloid fibers at the initial biofilm, but the staining might show false positive. We exposed the separated amyloid fibers to DNase I, Rnase and protease K for digestion, after that we could still observe integrated amyloid fibers. However, the initially added DNase I in BHIs can decrease the amount of amyloid fibers. The reason might be that planktonic *S. mutans* has rarely amyloid fibers and amyloid fibers are gradually formed during *S. mutans* biofilm formation. The initially added DNase can destroy eDNA, leading to fewer eDNA forming complex with amyloid fibers, and thus the amount of amyloid fibers are decreased. But once the amyloid fibers form complex with eDNA, the structure is resist to DNase (Gallo et al. 2015). Moreover, the combined use of EGCG and DNase in BHIs were more efficiently in destroying amyloid fibers and biofilm.

In conclusion, we firstly show the clear amyloid fibers in *S. mutans* and find two morphologies of amyloid fibers. Amyloid fibers exert important roles in *S. mutans* biofilm formation. Acidic PH and high temperature can accelerate the fibrillation process. Amyloid fibers form complex with eDNA in *S. mutans*, and combination use of DNase and EGCG is more efficient in decreasing the amount of amyloid fibers and biofilm biomass.

## Additional file

**Additional file 1. Figure S1:** The morphology of planktonic *S. mutans* observed by TEM. **Figure S2**: The characteristics of the extracted amyloid fibers.
