# Supplementary material for: Characteristics and influencing factors of amyloid fibers in S. mutans biofilm
Source: AMB Express. 2019 Feb 28;9:31. doi: 10.1186/s13568-019-0753-1 (PMC6395465; doi:10.1186/s13568-019-0753-1)
Supplement: Supplementary file 1 — Additional file 1. Figure S1: The morphology of planktonic S. mutans observed by TEM. Figure S2: The characteristics of the extracted amyloid fibers. [file 13568_2019_753_MOESM1_ESM.pdf]

Journal name: *AMB Express*

Manuscript Title: Characteristics and influencing factors of amyloid fibers in *S.mutans* biofilm

Dongru Chen, Yina Cao, Lixia Yu, Ye Tao, Yan Zhou, Qinghui Zhi, Huancai Lin*

Department of Preventive dentistry , Guanghua School of Stomatology, Guangdong Provincial Key Laboratory of Stomatology, Sun Yat-sen University, Guangzhou, Guangdong, China

*Corresponding author: Huancai Lin, email: linhc@mail.sysu.edu.cn; Tel: 020-83744816

Figure S1

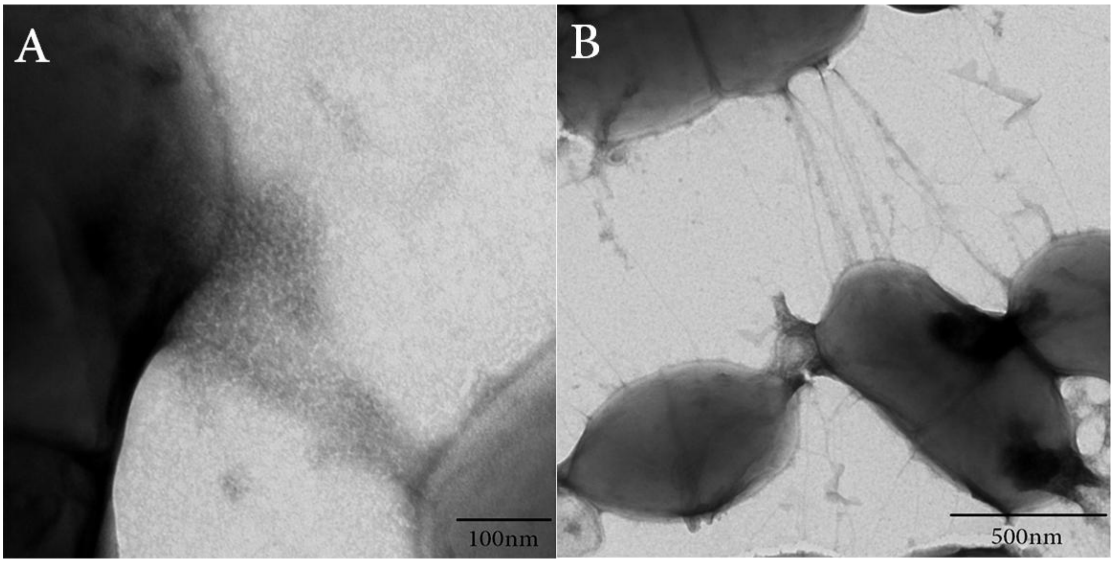

Figure S2

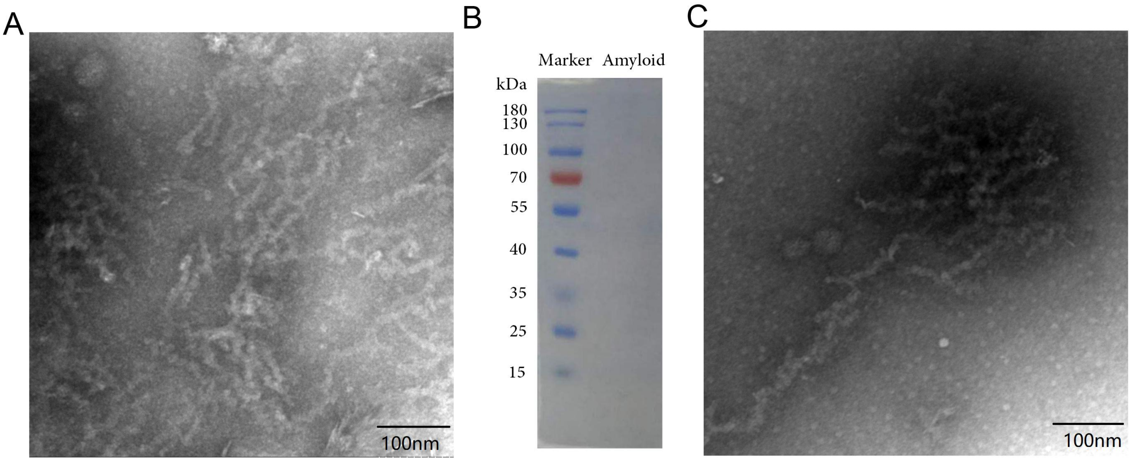

Supplementary Figure legends:

Figure S1: A: Amyloid fibers could also be observed in planktonic state, but with small amount. B: The main connection structures in planktonic *S.mutans*.

Figure S2: Characteristics of the extracted amyloid fibers. A: TEM image showed the successful isolation of amyloid fibers. B: The isolated amyloid fibers without any treatment were run by SDS-page, and no bands could be seen. C: TEM image of the extracted amyloid fibers after treated by protease K, DNase I and RNase.
